# ZIF-8-Based Nitrogen and Monoatomic Metal Co-Doped Pyrolytic Porous Carbon for High-Performance Supercapacitor Applications

**DOI:** 10.3390/nano14161367

**Published:** 2024-08-21

**Authors:** Xiaobo Han, Yihao Geng, Jieni Wang, Shuqin Zhang, Chenlin Wei, Leichang Cao, Shicheng Zhang

**Affiliations:** 1 Miami College, Henan University, Kaifeng 475004, China; 18625938707@163.com (X.H.); gyh618@henu.edu.cn (Y.G.); jieniwang@126.com (J.W.); zhangshuqin@henu.edu.cn (S.Z.); chenlinwei311@gmail.com (C.W.); 2 College of Chemistry and Molecular Sciences, Henan University, Kaifeng 475004, China; 3 Shanghai Key Laboratory of Atmospheric Particle Pollution and Prevention (LAP3), Department of Environmental Science and Engineering, Fudan University, Shanghai 200433, China; zhangsc@fudan.edu.cn

**Keywords:** zeolite imidazolate framework, nitrogen doped, transition metal, supercapacitor

## Abstract

Metal–organic frameworks (MOFs) receive wide attention owing to their high specific surface area, porosity, and structural designability. In this paper, ZC-Ru and ZC-Cu electrodes loaded with monatomic Ru and Cu doped with nitrogen were prepared by pyrolysis, ion impregnation, and carbonization process using ZIF-8 synthesized by static precipitation as a precursor. ZC-Cu has a high specific surface area of 859.78 m^2^ g^−1^ and abundant heteroatoms O (10.04%) and N (13.9%), showing the specific capacitance of 222.21 F g^−1^ at 0.1 A g^−1^ in three-electrode system, and low equivalent series resistance (Rct: 0.13 Ω), indicating excellent energy storage capacity and electrical conductivity. After 10,000 cycles at 1 A g^−1^ in 6 M KOH electrolyte, it still has an outstanding capacitance retention of 99.42%. Notably, symmetric supercapacitors ZC-Cu//ZC-Cu achieved the maximum power density and energy density of 485.12 W·kg^−1^ and 1.61 Wh·kg^−1^, respectively, positioning ZC-Cu among the forefront of previously known MOF-based electrode materials. This work demonstrates the enormous potential of ZC-Cu in the supercapacitor industry and provides a facile approach to the treatment of transition metal.

## 1. Introduction

The pervasive consumption of fossil fuels (e.g., coal, oil, and natural gas) has ignited a confluence of global environmental challenges, notably energy depletion and global warming, prompting a shift towards novel alternative energy avenues [1,2]. Although renewable energy sources have addressed escalating fossil fuel demands and pollution to some extent, their adoption remains hindered by inherent limitations such as instability, seasonality, discontinuity, and suboptimal conversion efficiencies [3,4,5]. Supercapacitors, as a new type of energy storage device, provide a promising method for the rapid storage of renewable energy and show a wide range of applications in new energy power generation systems, electronics, automotive transportation, and other fields [6,7,8]. To further the application of supercapacitors in the commercial market and the field of energy storage, it is urgent to develop and research devices with superior performance, environmental friendliness, and simplified structure [9,10].

Supercapacitors (SCs) are energy storage devices between traditional capacitors and batteries, mainly composed of electrodes, separators, and electrolytes [11]. Compared with traditional energy storage devices such as ordinary capacitors and batteries, supercapacitors have the advantages of fast charging and discharging, long life, high cycle stability, environmental friendliness, and high safety [12,13,14]. The three common basic electrode materials are carbon-based, metal oxides, and conductive polymers [15,16,17]. Among them, carbon-based materials are based on the energy storage process of accumulating charge on the electrode surface [18], and the latter two are Faraday reactions occurring on the electrode surface [19]. In general, maintaining a high power density while obtaining a high energy density requires the electrode material to have a suitable hierarchical porous structure and a high specific surface area [20]. Therefore, the study of energy storage active materials with stable structures, large specific surface areas, and well-developed pores has become a hot spot.

Metal–organic frameworks (MOFs), also known as porous coordination polymers [21], usually have a very high specific surface area and porous nanostructures which are conducive to exceptional electrochemical properties when used as electrode materials. MOF materials, with highly controllable pore structure and diverse chemical composition, can achieve pore size regulation and the introduction of functional groups and active sites by doping different metal ions and organic ligands, optimizing storage and transmission performance to meet the conductivity demands of electrochemical applications [22,23,24]. It has been proven that the nitrogen-doped polyhedron (MOF-NCP) derived from MOFs has a large specific surface area, adjustable pore size, and easy chemical modification [25]. However, pure MOF-NCP structures make it difficult to achieve efficient and rapid electron transport and electrolyte wetting [26,27]. The preparation of MOFs-based monatomic nitrogen-doped porous electrode materials by adding transition metal ions to MOFs has emerged as a new direction to be explored urgently. Zeolitic imidazolate framework-8 (ZIF-8) is a novel type of metal–organic framework (MOF) material, specifically, a porous crystal material composed of high nitrogen-doped imidazole rings, with Zn^2+^ as the central coordination ion [28]. Compared with other MOF materials, ZIF-8 has rich nitrogen content, which can add additional pseudocapacitance, generally considered to be a promising precursor template for electrode materials [29,30]. Kim et al. [31] prepared a hollow activated carbons (HACs) electrode material, by using zeolite imidazolate framework-8 (ZIF-8) as a precursor, improving the maximum specific capacitance and capacitance retention. Wang et al. [32] studied the electrochemical properties of MOF ZIF-8 annealed at 500 C, demonstrating that the Zn@ZIF-8-500 electrodes delivered a high energy density, a long life of 1600 cycles, and retained 72% capacity over 20,000 cycles. However, ZIF-8-derived carbon has considerable pore space, and few unsaturated metal centers, resulting in it being prone to collapse during pyrolysis and having a low electron adsorption affinity. The addition of transition metals [33] (such as Fe, Co, Ru, Cu, etc.) to replace part of Zn^2+^ in ZIF-8-derived carbon has been used as a feasible method to improve MOF-derived carbon materials. When low-valent metal ions (such as Fe^2+^, Co^2+^, Cu^2+^, Ru^3+^, etc.) coordinate with N-containing heterocyclic ligands (such as imidazole, pyrazole, etc.), the strengthening of coordination bonds helps to improve the overall stability of MOF-derived carbon materials, further strengthen the pore structure, and prevent pore collapse. MOF-derived carbon materials with higher crystallinity and more stable structure can be prepared by selecting suitable transition metal ions, organic ligands, and synthesis methods. With the characteristics of the adjustable structure, the MOF-derived carbon doped with transition metals shows the morphology coated by metal and organic ligands, which not only prevents carbon materials from collapsing after pyrolysis but enhances the electrochemical performance of the modified material [34].

In this work, we utilized ZIF-8 synthesized by static precipitation as a precursor for the preparation of ZC-Ru and ZC-Cu electrodes loaded with Ru^3+^ and Cu^2+^ doped with monatomic nitrogen via pyrolysis, ion impregnation, and the carbonization process. The electrochemical properties of different electrode materials ZC, ZC-Ru, and ZC-Cu were investigated. ZC-Cu showed high specific capacitance (222.21 F g^−1^ at a current density of 0.1 A g^−1^), low equivalent series resistance (Rct: 0.13 Ω), outstanding capacitance retention (99.42% after 10,000 cycles at a current density of 1 A g^−1^ in 6 M KOH), and excellent power density (485.12 W·kg^−1^) and energy density (1.61 Wh·kg^−1^). In addition to providing a new pathway for the treatment of transition metals, this study demonstrates the enormous potential of ZC-Cu in the supercapacitor industry, expanding our current understanding of the application of MOF-based materials in the field of electrochemistry.

## 2. Materials and Methods

### 2.1. Materials and Reagents

The zinc nitrate hexahydrate (Zn(NO_3_)_2_·6(H_2_O), AR), 2-methylimidazole (C_4_H_6_N_2_, AR), ruthenium chloride trihydrate (RuCl_3_·3H_2_O, AR), copper chloride dihydrate (CuCl_2_·2H_2_O, AR), acetylene black, and polytetrafluoroethylene (PTFE) were provided by Aladdin Biochemical Technology Co., Ltd., Shanghai, China. The other chemical reagents and materials used in this study included anhydrous ethanol (CH_3_CH_2_OH, AR) and nickel foam used to prepare the working electrodes, as well as the methyl alcohol (CH_3_OH, AR) as the auxiliary solvent procured from Sinopharm Chemical Reagent Co., Ltd., Shanghai, China. The equipment used in this study included a tubular furnace (CHY-1200, Henan Chengyi Equipment Technology Co., Ltd., Zhengzhou, China), and a press machine (YLJ-5T, Hefei Kejing Material Technology Co., Ltd., Hefei, China).

### 2.2. Preparation of ZIF-8-Based Nitrogen-Doped Porous Carbon

The preparation route of the sample is shown in Figure S1 (Supporting Information). A total of 2.975 g Zn(NO_3_)_2_·6(H_2_O) and 6.8 g 2-methylimidazole were dissolved in 113 mL of methanol, respectively, and stirred for 10 min after mixing these two solutions. Then, the white turbid liquid was stood for 24 h and the supernatant liquid was poured out. The precipitate was transferred to a centrifuge for centrifugation. The white precipitate after centrifugation was placed in an oven at 60 °C for 12 h to obtain a white solid (ZIF-8). A total of 2 g of white solid was weighed into a nickel crucible and pre-carbonized in a tube furnace under a nitrogen atmosphere. The temperature was raised to 800 °C at a heating rate of 3 °C min^−1^ and kept constant for 2 h to obtain a nitrogen-doped pre-carbonized product named ZC. In total, 0.2 g ruthenium trichloride trihydrate (RuCl_3_·3H_2_O) and 0.2 g copper chloride dihydrate (CuCl_2_·2H_2_O), which were dissolve in 1 mL methanol, respectively, denoted as solution A and solution B. Then, 1 g ZC was impregnated in solution A and solution B, respectively. The impregnated ZC was pyrolyzed under the same conditions for ZC preparation (800 °C, heating rate of 3 °C min^−1^ and kept constant for 2 h) to obtain the nitrogen and monoatomic metal co-doped porous carbon named as ZC-Ru and ZC-Cu, respectively.

### 2.3. Characterizations of Materials

Field emission scanning electron microscopy (SEM, JSM-7610F, JEOL, Tokyo, Japan) was used to study the morphology of the microscopic surface of the sample, and energy diffraction spectroscopy (EDS) was used to study the elemental composition. By X-ray diffraction (XRD, Bruker D8 Advance, Bruker, Ettlingen, Germany), the diffraction pattern was obtained in the range of 5–60° at the scanning rate of 2° min^−1^, and the crystal structures of all samples were determined and the crystallinity of the samples was analyzed. Raman spectra are collected on a Raman spectrometer (Renishaw in Via Reflex) to detect defects in the sample, with a laser wavelength of 532 nm. The elemental compositions and bonding states of the samples were characterized by X-ray photoelectron spectroscopy (XPS). The adsorption and desorption curves of N_2_ samples were measured and analyzed using an automated surface area and pore size analyzer (ASAP 2020 Microphysics). Among them, the specific surface area of multimolecular layer adsorption was calculated by the BET method. Through the Barrett–Joyner–Halend (BJH) method, the pore area and pore size distribution curves of the material can be obtained.

### 2.4. Electrochemical Measurements

Firstly, the asymmetric working electrode was prepared by mixing 80 wt% activated carbon, 10 wt% carbon black, and 10 wt% PTFE in anhydrous ethanol, coating onto a 1 × 1 cm^2^ nickel foam, and compressing at 15–20 MPa using a tablet press. For the two-electrode system, the active material was coated on a 1.1 cm diameter nickel foam disk. The basic electrochemical tests (e.g., cyclic voltammetry (CV), Constant-current Charge–discharge (GCD), and Electrochemical Impedance Spectroscopy (EIS) were performed using identical conditions and devices as in previous experiments [35,36]. The calculations of specific capacitance, power density, and energy density are also explained in detail in Supporting Information.

In order to further analyze the capacitance characteristics of the sample [37], dynamic analysis was performed at different scanning rates, and was calculated using Equation (1):

(1) $$i=av^{b}$$

where *i* (A) is the response current at different scanning rates, *v* (mV s^−1^) is the scanning rate, *a* and *b* are constants, where *b* represents the slope. Take the logarithm of both sides in the Equation (2):

(2) log⁡i=log⁡a+blog⁡v

The contribution ratio of capacitance was obtained using the Dunn method (Equation (3)):

(3) $$i\left(v\right)=k_{1}v+k_{2}v^{\frac{1}{2}}$$

Here, *i*(*v*) represents the real-time current of the CV test with a scan rate of *v*, and the current regulated by the surface control capacitor and the current regulated by the diffusion control capacitance are $k_{1}v$ and $k_{2}v^{\frac{1}{2}}$. To facilitate analysis and processing, Equation (3) is simplified to Equation (4):

(4) $$\frac{i\left(v\right)}{v^{\frac{1}{2}}}=k_{1}v^{\frac{1}{2}}+k_{2}$$

By determining the constant value of the $k_{1}$ and $k_{2}$, the surface control capacitance and diffusion control capacitance at different scanning speeds can be quantitatively calculated according to Equation (4).

## 3. Results and Discussion

### 3.1. Material Characteristics

**Figure 1:**
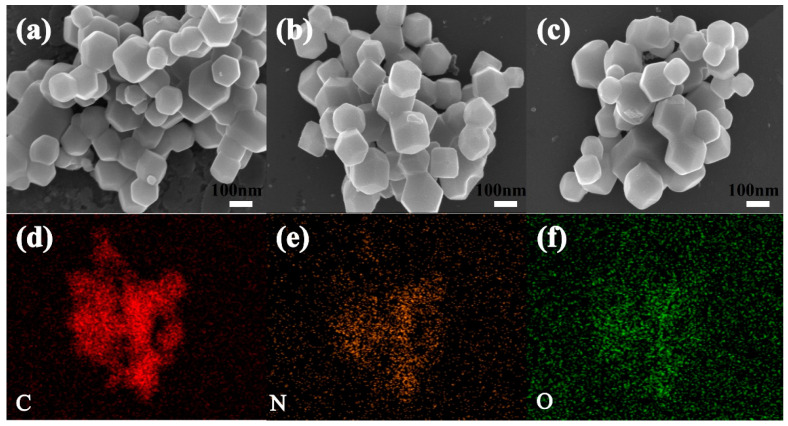
SEM images of (**a**) ZC, (**b**) ZC-Ru, (**c**) ZC-Cu, (**d**–**f**) EDS mapping of ZC-Cu.

To explore the microstructure of ZC, ZC-Ru, and ZC-Cu, SEM morphology analysis of the samples was performed, as shown in Figure 1a–c. It can be found that the regular crystalline polyhedral structure of ZIF-8 [38] is retained. In the process of carbonization, Zn^2+^ is reduced to Zn elemental, and the decomposition of NO_3_^−^ at high temperature causes the carbon material to be in situ doped with N element, thus retaining the developed pore structure and forming large pore channels and broken carbon fragments.

In the process of preparing ZIF-8 pyrolytic porous carbon, the nitrogen and oxygen derived from Zn(NO_3_)_2_·6(H_2_O) and some residual Zn(NO_3_)_2_·6(H_2_O) formed NO_2_, O_2,_ and other gases at high temperatures (the reaction mechanism is shown in Equation (5)), which promotes the development of micro/macro pores and forms porous structures in carbon materials. In addition, heteroatoms are also a key to increasing capacitance performance. As shown in Figure 1d–f, energy dispersive spectral analysis (EDS) was carried out on ZC-Cu. The results of element spectra showed that the distribution of C, N, O, and Cu (see Figure S2 and Table S1 (Supporting Information)) elements was uniform, indicating that the in situ self-doping of ZC-Cu was successful. Table 1 shows the specific surface area (SSA) of ZC, ZC-Ru, and ZC-Cu are 490.08 m^2^ g^−1^, 692.54 m^2^ g^−1,^ and 859.78 m^2^ g^−1^, respectively. ZC-Cu shows the highest SSA with a total pore volume of 0.49 cm^3^ g^−1^, indicating that ZC-Cu has excellent layered porosity.

(5) $$2Zn{\left(NO_{3}\right)}_{2}\cdot 6H_{2}O\to 2ZnO+4NO_{2}+O_{2}\;12H_{2}O$$

**Figure 2:**
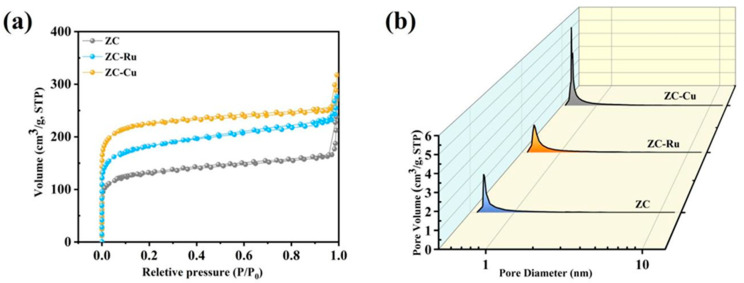
(**a**) Nitrogen adsorption–desorption isotherms of ZC, ZC-Ru, and ZC-Cu, (**b**) pore size distributions of ZC, ZC-Ru, and ZC-Cu.

As shown in Figure 2, SSA and pore structure distribution of the samples were analyzed by N_2_ adsorption and desorption curves. Figure 2a,b show the N_2_ adsorption isotherm and pore size distribution of all samples. As can be seen from Figure 2a, ZC, ZC-Ru, and ZC-Cu show the combination of type I and type IV isotherms, indicating the presence of more micropores in the material. When the relative pressure (P/P_0_) was low (P/P_0_ < 0.05) [39], it was found that the adsorption capacity increased rapidly, indicating that there were abundant micropores in the sample. However, when the relative pressure was higher (P/P_0_ > 0.4), the hysteresis effect occurred and the H4 hysteresis ring was generated, indicating the existence of typical microporous and mesoporous structures. Figure 2b shows the pore size distribution, and it is found that all samples have stratified microporous (1–2 nm) and micromesoporous (3–4 nm) structures. Micromesopores can provide larger specific surface area and pore volume [40] and increase the number of active sites. Micromesopores can promote the rapid diffusion of ions and the rapid transfer of substances.

**Figure 3:**
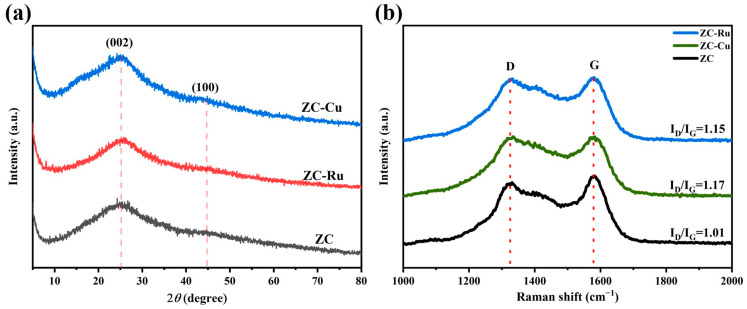
(**a**) XRD patterns of ZC, ZC-Ru, and ZC-Cu, (**b**) Raman spectra of ZC, ZC-Ru, and ZC-Cu.

The XRD pattern can be used to analyze the crystallinity of carbon materials, and the XRD pattern of each sample is shown in Figure 3a. It can be found that diffraction peaks near 25° and 45° are generated by the (002) and (100) lattice planes. This indicates the presence of amorphous carbon [41] and graphitized structures in the sample. The interlayer spacing (002) and plane spacing (100) crystal planes show graphitic carbon [42] and interlayer condensation, and no additional peaks are found in the sample. In the small Angle region (2θ < 10°), the obvious high-intensity peak indicates that the sample contains a large number of micropores.

The Raman spectrum of the sample is shown in Figure 3b, and two obvious characteristic peaks are observed. They represent the D-band (1332 cm^−1^) caused by graphite structural defects [43] and the G-band (1578 cm^−1^) generated by the in-plane tensile vibrations of sp2 hybrid carbon [44] in graphite crystals. The intensity ratio (I_D_/I_G_) between the G-band and D-band [41] was used to evaluate the degree of graphitization of the sample. After calculation, the I_D_/I_G_ values of ZC, ZC-RU, and ZC-Cu are 1.01, 1.15, and 1.17, respectively. A larger I_D_/I_G_ ratio means that there is a higher proportion of defective or amorphous carbon structures in the sample relative to ordered graphite or graphene structures. In other words, the lower the degree of graphitization of the material, the more abundant the defects. It was found that the I_D_/I_G_ value of ZC-Cu was higher, which enabled the material to better adsorb electrolyte ions and thus enhance the specific capacitance performance.

**Figure 4:**
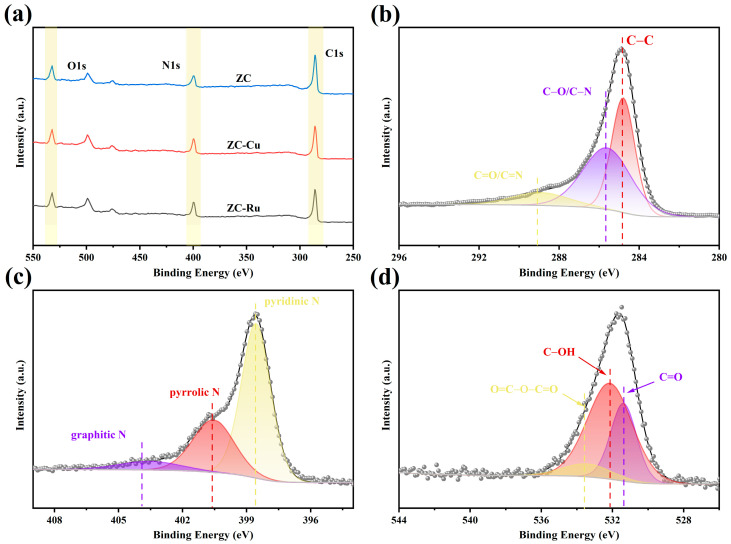
(**a**) XPS full spectrum of ZC, ZC-Ru, and ZC-Cu. (**b**) XPS high-resolution spectrum of C1s. (**c**) XPS high-resolution spectrum of N1s. (**d**) XPS high-resolution spectrum of O1s.

The XPS was used to further investigate the elemental composition and bonding states of the sample. Therefore, the XPS spectrum of the sample is shown in Figure 4a. The XPS spectra of ZC-Cu were further studied, and typical characteristic peak signals of C1s, O1s, and N1s were observed from the measured spectra, showing the presence of C (70.7%), abundant heteroatoms O (10.99%) and N (18.31%). The contents of carbon, oxygen, and nitrogen in other samples were summarized in Table S2 (Supporting Information). The peak values of the high-resolution C1s spectra of ZC-Cu (Figure 4b) are 284.80 eV, 285.60 eV, and 288.93 eV, respectively, which are caused by C-C (sp^2^ hybrid) C-N/C-O and C=C/C=N groups. The presence of C-N groups [45] indicates the in situ self-doping of nitrogen. The spectrum of N1s (Figure 4c) can be decomposed into three distinct peaks: 398.56 eV, 400.54 eV, and 403.52 eV, corresponding to pyridinic N, pyrrolic N, and graphitic N. Among them, the nitrogen atoms in pyridine nitrogen (62.66%) can act as electron-withdrawing groups, creating local electron defects or “holes” in the carbon material, which can be used to store charges and increase the capacitance of the material. Pyrrolidine (30%) can act as an electron donor group [46], producing “delocalized electrons” in the carbon material, and the excess electrons produced can make the material more conductive and reduce charge transfer resistance. The presence of graphitic nitrogen (7.34%) implies the formation of a graphitized framework that accelerates the transfer of electrons. Three peaks appear in the spectrum of high-resolution O1s (Figure 4d), and it can be found that the oxygen group components mainly exist in the carbon skeleton in the form of C=O (531.37 eV), C-OH (532.15 eV), and O=C-O-C=O (533.44 eV) bonds.

### 3.2. Electrochemical Properties in a Three-Electrode System

**Figure 5:**
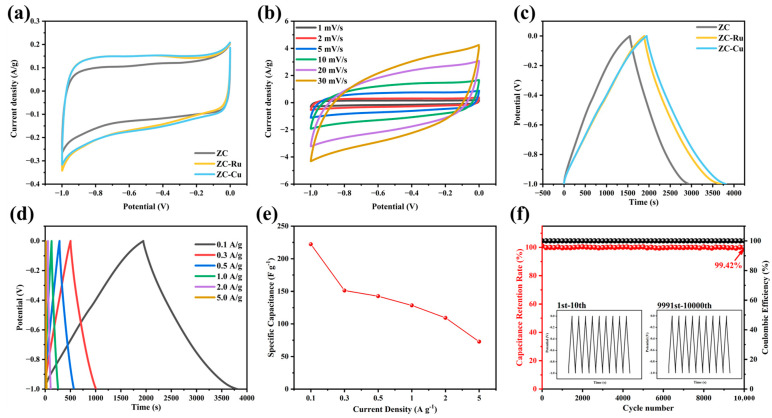
(**a**) CV curves of ZC, ZC-Ru, and ZC-Cu. (**b**) CV curves of ZC-Cu at different scanning rates from 1 to 30 mV s^−1^. (**c**) GCD curves of ZC, ZC-Ru, and ZC-Cu. (**d**) GCD curves of ZC-Cu at different densities from 0.1 to 5 A g^−1^. (**e**) Specific capacitances of the ZC-Cu electrodes (**f**) Cycling performance of ZC-Cu at 1 A g^−1^ for 10,000 cycles.

To investigate the potential usefulness and practical value of the fabricated electrode materials, the electrochemical performance of the three-electrode system was tested. The cyclic voltammetry (CV) test was carried out at a scanning rate of 1 mV s^−1^ in a 6 M KOH electrolyte, and the CV curves of the working electrodes were obtained, as shown in Figure 5a. It was found that the CV curves of the samples were all quasi-rectangular. Compared with ZC and ZC-Ru, ZC-Cu had the largest rectangular area and exhibited the best capacitance performance (see Equation (S1) (Supporting Information)). No significant bending was observed in the CV curve. This indicates the charge diffusion dynamics and good reversibility [47]. To have a more comprehensive understanding of the electrochemical performance of the ZC-Cu electrode material, CV tests of the ZC-Cu electrode material at different scanning rates were carried out, as shown in Figure 5b. Next, the GCD curve, specific capacitance, and capacitance retention further reflect the excellent electrochemical behavior of ZC-Cu electrode materials. The GCD curve is shown in Figure 5c, and the curves are roughly symmetrical isosceles triangles. It further exhibits typical double-layer capacitor behavior [48] and good reversibility of charge and discharge. ZC-Cu has a longer charge–discharge time, indicating the maximum specific capacitance, which is the same as the CV curve analysis results. Figure 5d shows that all GCD curves have similar triangles as the current density increases. When the current density is 0.1 A g^−1^, the specific capacitance of ZC-Cu is 222.21 F g^−1^. Even with A 50-fold increase in current density to 5 A g^−1^, the specific capacitance of ZC-Cu still reaches 72.77 F g^−1^ and the capacity retention rate reaches 32.8% (Figure 5e), which is higher than the other two electrode materials. ZC-Cu shows good magnification performance [49] and capacitance performance. In order to test the cyclic stability of ZC-Cu, after 10,000 cycles with A current density of 1 A g^−1^ (Figure 5f), the capacitance retention rate is 99.42% (Equation (S9) (Supporting Information)) and the capacity attenuation is negligible, showing excellent cyclic stability and its reversible and efficient charge–discharge behavior.

Previous studies have shown [50] that the capacitive performance of SCs is determined by two key factors, namely the degree of pore development (i.e., V_micro_, V_micro_/V_pore_, and S_BET_) and nitrogen content. After repeated experiments and fitting the mean value of the results, we found similar conclusions. As displayed in Figure S3 (Supporting Information), it provides a linear fit of the capacitive properties of the sample to its potential influencing factors. It can be seen from the figure that V_micro_, V_micro_/V_pore_, and S_BET_ are all positively correlated with the specific capacitance size, and the correlation coefficients are all relatively high. However, the correlation coefficient between nitrogen content and specific capacitance is small, indicating that the degree of pore development is the main factor affecting the specific capacitance. These phenomena show that although the degree of pore development plays a dominant role in the capacitive properties of the prepared in situ nitrogen-doped porous carbon, it is not a single linear relationship. The capacitive performance is still determined by the combined effect of pore development [51] and N content. Among all the samples, ZC-Cu has the largest specific capacitance, which can be attributed to its excellent SSA (859.78 m^2^ g^−1^) and total pore volume (0.49 cm^3^ g^−1^), forming a unique layered porous structure that provides more electrolyte ion adsorption sites. Abundant micropores and mesoporous pores [52] enhance the adsorption capacity and diffusion rate of ions.

**Figure 6:**
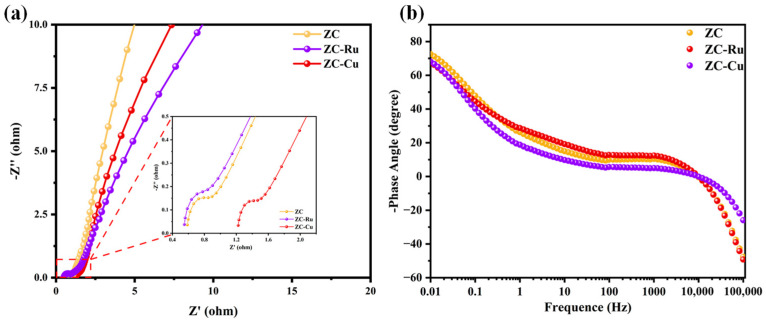
(**a**) Nyquist curves of ZC, ZC-Ru, and ZC-Cu, (**b**) BODE curves of ZC, ZC-Ru, and ZC-Cu.

In addition, EIS is used to study ion diffusion and charge transfer of different electrode materials, and Figure 6a and Figure S4 show the Nyquist diagram and EIS simulation equivalent circuit of the samples in the frequency range of 0.01 Hz to 100 kHz. Among them, the semicircle radius of the high-frequency region corresponds to the charge transfer resistance R_ct_ [53], and the intercept between the semicircle and the X-axis gives the electrolyte resistance R_s_. Based on the equivalent circuit fitting calculation, the R_ct_ corresponding to ZC, ZC-Ru, and ZC-Cu samples showed a trend of first increasing and then decreasing, which were 0.16 Ω, 0.70 Ω and 0.13 Ω, respectively, and the R_ct_ of electrode material ZC-Cu was the smallest. The line in the low-frequency region of the EIS curve corresponds to R_s_, and its value is inversely proportional to the slope of the line. The linear approximate slope of the sample in the low-frequency region is approximately 45°, indicating that the prepared carbon materials have ideal double-layer capacitance characteristics, mainly due to the existence of an internal porous structure, which provides a transport channel for ion diffusion. The ZC-Cu electrode material in the Nyquist curve had a smaller semi-circle and a steeper straight line. This exhibits higher electrical conductivity [54], with better rate capability and capacitance performance. In addition, the Bode curve of the electrode material is shown in Figure 6b. The phase angles of the three samples ZC, ZC-Ru, and ZC-Cu are 72.6°, 67.6°, and 68.1°, respectively, which are close to the ideal phase angles.

**Figure 7:**
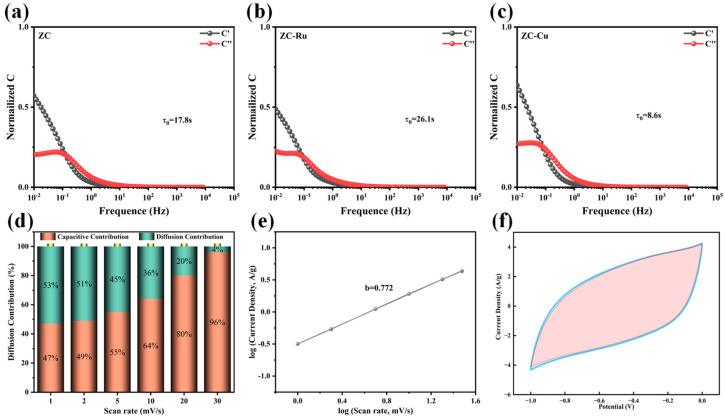
(**a**–**c**) The normalized real capacitance ($C^{\prime }(\omega )$) and imaginary capacitance ($C^{\prime \prime }(\omega )$) of ZC, ZC-Ru, and ZC-Cu, respectively. (**d**) The normalized ratios of capacitive contribution and diffusion contribution for ZC-Cu. (**e**) Plots of log (current density) vs. log (scan rate) for ZC-Cu. (**f**) Area plot of pseudocapacitance contribution of ZC-Cu electrode at a scan rate of 30 mV s^−1^.

According to the EIS data, the relationship between capacitance and frequency is analyzed by model $C\left(\omega \right)=C^{\prime }\left(\omega \right)+jC^{\prime \prime }(\omega )$. The relaxation time constant $\tau_{0}$ is used to estimate the charge–discharge rate [55] and can be obtained from the characteristic frequency $f_{0}$ and the imaginary part $C^{\prime \prime }(\omega )$ (Equations (S6) and (S7) (Supporting Information)). The values of the $\tau_{0}$ of ZC, ZC-Ru, and ZC-Cu electrodes were 17.8, 26.1, and 8.6 s, respectively (Figure 7a–c). The real part value $C^{\prime }\left(\omega \right)$ at low frequencies represents the maximum capacity of the electrode at constant current discharge. The smaller relaxation time enables ZC-Cu to achieve a faster ionic diffusion rate.

To determine the specific proportion of Faraday capacitance in the ZC-Cu material, we used the Dunn method for further quantitative analysis to determine the proportion of the surface capacitance control process (EDLC) and diffusion control process (pseudocapacitance) in the capacitance contribution of the three electrode materials. We further analyzed the CV curve and calculated the capacitance contribution rate at different scanning speeds according to Equations (3) and (4). The calculation results are shown in Figure 7d,f. The results show that the contribution of surface capacitance control accounts for most of the dynamic process. When the scanning rate is low, the contribution of heteroatoms to the pseudocapacitance is high. However, at scan rates up to 30 mV s^−1^, the surface-control capacitance contributes more than 95%, which is consistent with the high magnification capability demonstrated by the material. To further analyze the dynamic characteristics of the capacitor control behavior, the charge and discharge dynamics are qualitatively evaluated according to Equations (1) and (2). In addition, we investigated the logarithmic relationship between current density and scan rate by quantitatively distinguishing the contribution of capacitance to the current response (Figure 7e). The value between 0.5 and 1.0 [56] indicates that the charge storage of the electrode is regulated by a combination of surface control and diffusion control mechanisms. When b = 0.5, it indicates that charge storage is governed by the diffusion control process and shows the characteristics of the pseudocapacitor behavior of the battery or volume phase. In other words, the redox reaction occurs due to the insertion/withdrawal of electrolyte ions into the material and is characterized by slow reaction kinetics. When b = 1, it exhibits pure surface-controlled capacitance behavior, including double-layer capacitance and fast reversible pseudocapacitance occurring on the electrode surface, representing a fast electrochemical dynamic process. By linear fitting of the logarithm of current density and logarithm of scanning rate of ZC-Cu electrode, the obtained b value is 0.772, indicating that the charge storage of ZC-Cu electrode is mainly dominated by surface control process. Through the above analysis, we can more clearly understand the contribution ratio of different capacitor control processes in ZC-Cu materials, as well as the behavior characteristics of capacitors under different dynamic conditions.

### 3.3. Electrochemical Properties in a Symmetric Electrode System

**Figure 8:**
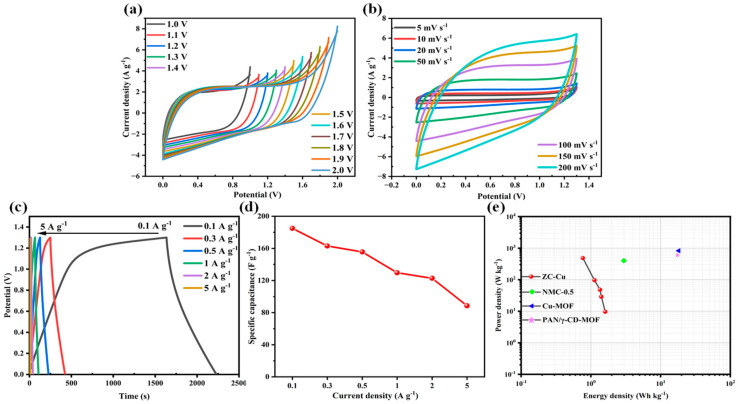
(**a**) CV of ZC-Cu at different open circuit voltages. (**b**) CV of ZC-Cu at 1.3 V at open-circuit voltage. (**c**) GCD of ZC-Cu at different current densities in the voltage window range of 0–1.3 V. (**d**) Specific capacitance of ZC-Cu at different scan rates in the voltage window range of 0–1.3 V. (**e**) Relationship between power density and energy density of ZC-Cu.

To evaluate the performance of ZC-Cu in practical applications, we used ZC-Cu as the electrode active material to assemble a symmetrical supercapacitor ZC-Cu//ZC-Cu and tested its performance in a two-electrode system with 6 M KOH as the electrolyte. CV tests were performed on ZC-Cu-based symmetric SCs in a 0–2 V voltage window with a scan rate of 100 mV s^−1^ (as shown in Figure 8a). When the voltage window is increased to 1.3 V, the anode current begins to appear in the CV curve [57], indicating that the symmetric SCs can work stably in the voltage window range of 0–1.3 V. CV and GCD tests were performed on ZC-Cu//ZC-Cu in the voltage window of 0–1.3 V. As shown in Figure 8b, the CV curve basically maintains a rectangular shape at different scanning rates, indicating good capacitive properties of the material. The GCD curve in Figure 8c presents an isosceles triangle shape at different current densities. When the current density is 0.1 A g^−1^, the specific capacitance of ZC-Cu//ZC-Cu is 184.97 F g^−1^. The variation curve of the specific capacitance of ZC-Cu with the current density can be seen in Figure 8d. To observe the overall performance of ZC-Cu as a precursor of electrode material, the relationship between energy density and power density was calculated by Equations (S4) and (S5) (Supporting Information) according to the GCD curve (Figure 8e). It can be found that with the increase in power density, the energy density decreases slightly, and the highest power density and energy density reach 485.12 W·kg^−1^ and 1.61 Wh·kg^−1^. This once again demonstrates the excellent electrochemical properties of ZC-Cu and further validates its potential for practical applications. Compared with other electrode materials in this category (Table 2), ZC-Cu electrode materials show outstanding cycling performance and excellent specific surface area, but there are still shortcomings in specific capacitance and energy storage. In the future, for ZIF-derived porous carbon electrode materials, in-depth research can be carried out on material structures (such as hollow or multistage pore structures), other composite materials (such as graphene, carbon nanotubes, etc.), and preparation process optimization to enhance competitiveness.

## 4. Conclusions

In summary, we successfully synthesized the ZC-Ru and ZC-Cu electrode materials from ZIF-8 prepared by static precipitation as the precursor via pyrolysis, ion impregnation, and carbonization process. The ZC-Cu electrode exhibited a high specific surface area of 859.78 m^2^ g^−1^ and abundant heteroatoms O (10.04%) and N (13.9%), delivering a specific capacitance of 222.21 F g^−1^ at the current density of 0.1 A g^−1^ and excellent conductivity and cyclic stability. Meanwhile, the symmetric ZC-Cu//ZC-Cu device achieved an ultrahigh specific capacitance of 184.97 F g^−1^ at 0.1 A g^−1^ current density and displayed robust charge–discharge capabilities, reaching a power density of 485.12 W·kg^−1^ and an energy density of 1.61 Wh·kg^−1^ at 30 A g^−1^. This work demonstrates the immense potential of ZIF-8-based nitrogen-doped pyrolytic nanoporous carbon as an electrical energy storage material and provides a facile and feasible way for transition metal treatment.

## Figures and Tables

**Table 1:** Porosity parameters of the ZC, ZC-Ru, ZC-Cu.

Samples	S_BET_ ^a^ (m^2^ g^−1^)	S_micro_ ^b^ (m^2^ g^−1^)	V_pore_ ^c^ (cm^3^ g^−1^)	V_micro_ ^d^ (cm^3^ g^−1^)	V_meso_ ^e^ (cm^3^ g^−1^)
ZC	490.08	347.35	0.42	0.14	0.25
ZC-Ru	692.54	508.97	0.43	0.20	0.19
ZC-Cu	859.78	712.33	0.49	0.28	0.17

^a^ BET surface area; ^b^ Micropore (<2 nm) surface area calculated; ^c^ Total pore volume obtained at P/P_0_ = 0.99; ^d^ Micropore volume calculated by t-plot method; ^e^ Mesopore volume calculated by BJH method.

**Table 2:** The comparison table of the overall properties of different electrode materials.

Electrode Materials	SSA (m^2^ g^−1^)	Specific Capacitance (F g^−1^)	Current Density (A g^−1^)	Energy Density (Wh kg^−1^)	Power Density (W kg^−1^)	Cycle Stability (%)	References
ZC-Cu	859.78	222.12	0.1	1.61	485.12	99.42/10,000 cycles	This work
Au@NCNC (nanocarbon-based material)	420.8	168.6	1	--	--	97/5000 cycles	[40]
NMC-0.5 (ZIF-derived mesoporous carbon)	2088	263	1	2.97	400	96.07/10,000 cycles	[58]
Cu-MOF	1321	104.8	0.5	18.2	825	87/10,000 cycles	[34]
PAN/γ-CD-MOF	134.7	283.3	0.5	17.5	600	97.5/6000 cycles	[59]

## Data Availability

Data are contained within the article and Supplementary Materials.

## Supplementary Materials

The following supporting information can be downloaded at: https://www.mdpi.com/article/10.3390/nano14161367/s1, Supplementary data associated with this article can be found in the file of Supporting Information. Refs. [43,60,61] are cited in the supplementary materials.
