# Supplementary material for: ZIF-8-Based Nitrogen and Monoatomic Metal Co-Doped Pyrolytic Porous Carbon for High-Performance Supercapacitor Applications
Source: Nanomaterials (Basel). 2024 Aug 21;14(16):1367. doi: 10.3390/nano14161367 (PMC11357066; doi:10.3390/nano14161367)
Supplement: Supplementary file 1 [file nanomaterials-14-01367-s001.zip › nanomaterials-3142949-supplementary.pdf]

Supporting Information

ZIF-8-Based Nitrogen and Monoatomic Metal Co-Doped Pyrolytic Porous Carbon for High-Performance Supercapacitor Applications

Xiaobo Han [1], Yihao Geng [1], Jieni Wang [1,2], Shuqin Zhang [1,2], Chenlin Wei [1,2], Leichang Cao [1,2,*] and Shicheng Zhang [3]

[1] Miami College, Henan University, Kaifeng 475004, China; 18625938707@163.com (X.H.);

gyh618@henu.edu.cn (Y.G.); jieniwang@126.com (J.W.); zhangshuqin@henu.edu.cn (S.Z.); chenlinwei311@gmail.com (C.W.)

[2] College of Chemistry and Molecular Sciences, Henan University, Kaifeng 475004, China

[3] Shanghai Key Laboratory of Atmospheric Particle Pollution and Prevention (LAP3), Department of Environmental Science and Engineering, Fudan University, Shanghai 200433, China; zhangsc@fudan.edu.cn

* Correspondence: clch666@henu.edu.cn

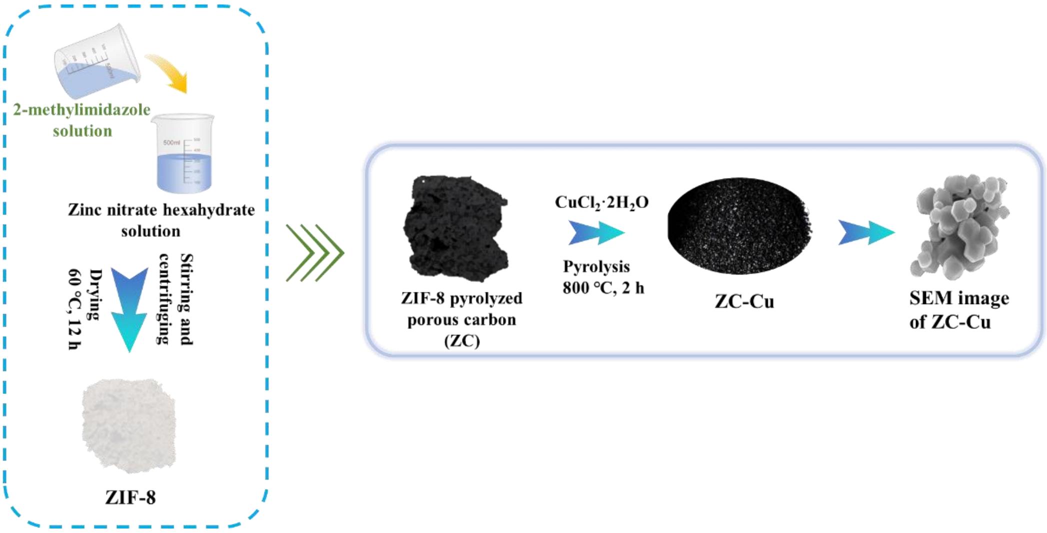

Figure S1. Synthetic route of ZC-Cu derived ZIF-8.

Electrochemical Measurements

In the three-electrode system, the calomel electrode was used as the reference electrode, the platinum wire electrode was used as the opposite electrode, and the prepared electrode sheet was used as the working electrode. The electrochemical testing was performed in a solution of 6 M KOH. EIS tests were performed on the working electrode at an open circuit voltage with an AC amplitude of 5 mV and a frequency range of 0.01Hz to 100kHz. The voltage working window of CV was set as -1 to -0 V and carried out at different scanning rates (1-30 mV s^{-1}), and the specific capacitance (F g^{-1}) can be calculated from the CV curves:

$$C_{sp} = \frac{A}{m \times s \times \Delta V} \quad \text{(S1)}$$

Where C_{sp} (F g^{-1}) is the specific capacitance, ΔV (V) is the voltage window of the working electrode, A represents half of the integrated area of the CV curve, m is the mass of the active material, and s is the scanning rate.

The voltage operating window of the GCD was set to -1 to -0V and charged and discharged at different current densities (0.1-5 A g^{-1}). The specific capacitance of a single electrode [1] (C_d, F g^{-1}) can also be obtained by the following Equation:

$$C_d = \frac{I\,\Delta t}{m\,\Delta V} \quad \text{(S2)}$$

In the two-electrode system, the voltage window for CV and GCD was set at 0-1.3V. The specific capacitance of a symmetric capacitor [2] can be obtained from Equations S3. The energy density E (Wh·kg^{-1}) and power density P (W·kg^{-1}) of the capacitor can also be obtained by using the following Equation:

$$C_t = 4 * \frac{I\,\Delta t}{m\,\Delta V} \quad \text{(S3)}$$

$$E = \frac{1}{8} \times C_d \times (\Delta V)^2 \times \frac{1}{3.6} \quad \text{(S4)}$$

$$P = \frac{E}{\Delta t} \times 3600 \quad \text{(S5)}$$

In order to further analyze the capacitor, the real and imaginary parts of the capacitor can be obtained from the following Equation:

$$C''(\omega) = \frac{Z'(\omega)}{\omega\,|Z(\omega)|^2} \quad \text{(S6)}$$

$$C'(\omega) = \frac{-Z''(\omega)}{\omega\,|Z(\omega)|^2} \quad \text{(S7)}$$

Where $Z(\omega)$ Represents the complex impedance ($\omega = 2\pi f$), $Z'(\omega)$ represents the real part, $Z''(\omega)$ represents the imaginary part.

Using the peak position of the peak position of the imaginary part of the complex capacito1r, the relaxation time constant (τ_0) can be calculated with the Equation, $\tau_0 = \frac{1}{f_0}$where f_0 is the frequency corresponding to the peak position.

Coulomb efficiency and cycle retention [3] can be derived from Equations S12 and S13:

$$CE = \frac{Q_d}{Q_c} = \frac{I_{discharge} \times t_{discharge}}{I_{charge} \times t_{charge}} \quad \text{(S8)}$$

Cyclic retention (%) after nth cycle = $\frac{specific\ capacitance\ at\ n^{th}\ cycle}{specific\ capacitance\ at\ 1^{st}\ cycle} \times 100\%$ (S9)

Where Q represents the amount of charging. For a constant current, Q = It where I

represents current, and t represents time.

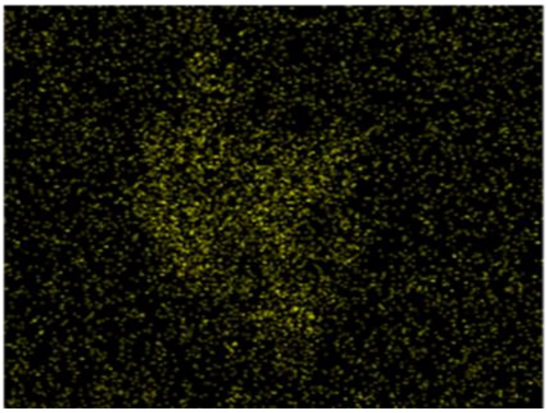

Figure S2. EDS mapping of ZC-Cu.

Table S1. The element distribution of the total spectrum diagram of ZC-Cu.

**Distribution diagram of total spectrum diagram**				
**Element**	**Line type**	**Weight percent**	**Wt % Sigma**	**atomic percent**
C	K type	52.55	1.08	60.36
O	K type	19.28	0.80	16.63
Zn	K type	5.05	0.56	1.03
N	K type	22.00	1.52	21.67
Cu	K type	1.12	0.44	0.32
Total		100.00		100.00

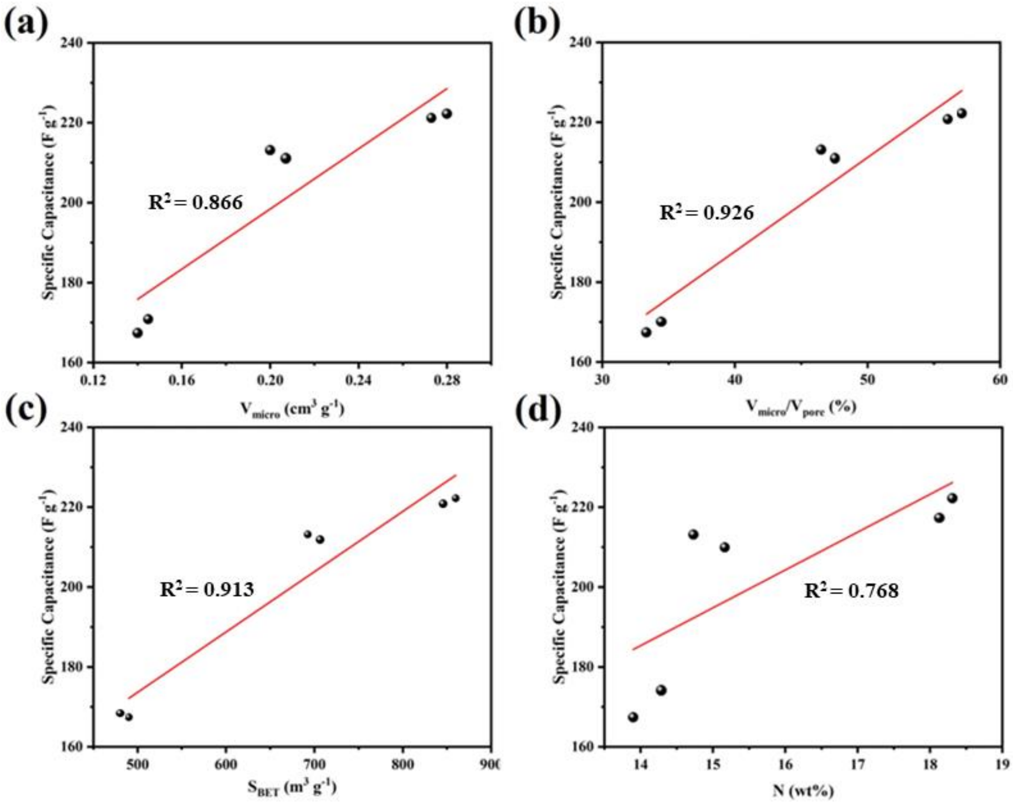

Figure S3. Linear fit of capacitance performance to potential contributing factors. (a) The relationship between capacitance performance and V_{micro} (cm^3 g^{-1}). (b) The relationship between capacitance performance and V_{micro}/V_{pore} (%). (c) The relationship between capacitance performance and S_{BET} (m^2 g^{-1}). (d) The relationship between capacitance performance and nitrogen content (wt%).

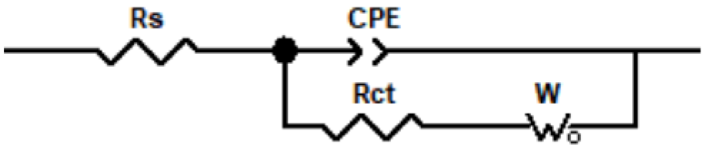

Figure S4. EIS simulation equivalent circuit.

Table S2. The carbon, oxygen, and nitrogen content of all samples.

Sample	Elemental composition		
	C (wt%)	O (wt%)	N (wt%)
ZC	76.07	10.04	13.90
ZC-Ru	75.63	9.64	14.73
ZC-Cu	70.70	10.99	18.31

References

1. Karnan, M.; Subramani, K.; Sudhan, N.; Ilayaraja, N.; Sathish, M. *Aloe vera* Derived Activated High-Surface-Area Carbon for Flexible and High-Energy Supercapacitors. *Acs Applied Materials & Interfaces* **2016**, *8*, 35191-35202, doi:10.1021/acsami.6b10704.
2. Yang, P.; Mai, W. Flexible solid-state electrochemical supercapacitors. *Nano Energy* **2014**, *8*, 274-290, doi:10.1016/j.nanoen.2014.05.022.
3. Rahman, M.M.; Hossen, M.R.; Alam, I.; Rahman, M.H.; Faruk, O.; Nurbas, M.; Rahman, M.M.; Khan, M.M.R. Synthesis of hexagonal boron nitride based PANI/h-BN and PANI-PPy/h-BN nanocomposites for efficient supercapacitors. *Journal of Alloys and Compounds* **2023**, *947*, doi:10.1016/j.jallcom.2023.169471.
